# Alpha Synuclein only Forms Fibrils In Vitro when Larger than its Critical Size of 70 Monomers

**DOI:** 10.1002/cbic.202100285

**Published:** 2021-08-24

**Authors:** Santiago Enrique Sanchez, Daniel R. Whiten, Georg Meisl, Francesco Simone Ruggeri, Eric Hidari, David Klenerman

**Affiliations:** ^1^ Department of Chemistry University of Cambridge Lensfield Road Cambridge CB2 1EW UK; ^2^ Stanford Cancer Institute Stanford University School of Medicine Stanford CA USA; ^3^ Kolling Institute of Medical Research University of Sydney Australia; ^4^ Laboratories of Organic and Physical Chemistry Wageningen University and Research Stippeneng 4 6708 WE Wageningen The Netherlands; ^5^ UK Dementia Research Institute at Cambridge Cambridge CB2 0XY UK

**Keywords:** α-synuclein, fibrils, protein aggregation, single molecule AFM, single molecule fluorescence

## Abstract

The aggregation of α‐synuclein into small soluble aggregates and then fibrils is important in the development and spreading of aggregates through the brain in Parkinson's disease. Fibrillar aggregates can grow by monomer addition and then break into fragments that could spread into neighboring cells. The rate constants for fibril elongation and fragmentation have been measured but it is not known how large an aggregate needs to be before fibril formation is thermodynamically favorable. This critical size is an important parameter controlling at what stage in an aggregation reaction fibrils can form and replicate. We determined this value to be approximately 70 monomers using super‐resolution and atomic force microscopy imaging of individual α‐synuclein aggregates formed in solution over long time periods. This represents the minimum size for a stable α‐synuclein fibril and we hypothesis the formation of aggregates of this size in a cell represents a tipping point at which rapid replication occurs.

## Graphical abstract

**The critical size** for α‐synuclein to form a stable fibrils, which can then replicate by monomer addition and fragmentation, has been found to be 70 monomers. Preventing aggregates from reaching this critical size would be a key cellular protective mechanism.

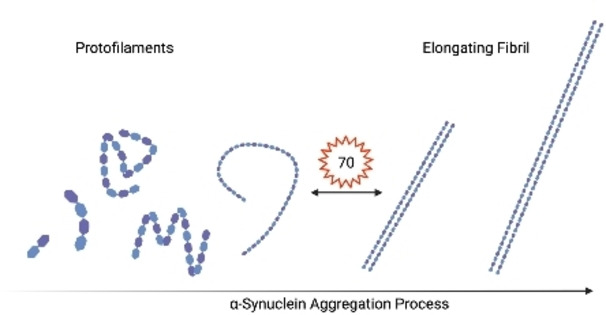

## Introduction

A body of data has shown how intrinsically disordered proteins such as beta amyloid, tau and α‐synuclein aggregate in the test‐tube and the rate constants for the key molecular steps have recently been determined.[^1^, ^2^, ^3^, ^4^, ^5^, ^6^] α‐Synuclein monomers have been shown to rapidly form small globular proteinase‐K‐sensitive aggregates by monomer addition which then undergo a slow structural conversion to fibrillar proteinase‐K‐resistant aggregates, that then can rapidly grow into fibrils by further addition of monomers.[^1^, ^6^] Experiments over longer times have shown that these fibrils can fragment into smaller aggregates and hence replicate in the test‐tube^5^. It is speculated that a similar process takes place in neurons in the brain and leads to the spreading of α‐synuclein from neuron to neuron in a mechanism often referred to as prion‐like spreading.^[4]^

The formation of fibrillar aggregates from a solution of monomeric proteins generally involves three types of processes; primary nucleation, which forms the initial aggregates directly from monomer; elongation, which grows existing aggregates by addition of monomers from solution; and multiplication, which increases the number of aggregates, for example by fragmentation or secondary nucleation on the surface of existing aggregates.^[7]^ A key parameter to describe an aggregating system is its rate of replication, κ, whose inverse is proportional to the time it takes to double the number of fibrils. It can be shown that quite generally this replication rate is determined by the product of the elongation and multiplication rates.^[8]^ Through measurements of the aggregation of purified α‐synuclein, the rate constants for elongation and fragmentation of fibrillar aggregates of α‐synuclein have recently been determined.^[4]^ However, it is not known how large an aggregate needs to be before conversion from a pre‐fibrillar to intermediate and mature cross‐β fibrillar forms is favorable. This quantity is important since it defines the minimum size of a replication‐competent fibrillar aggregate. If this size is large then fragmentation is more likely to produce an aggregate below this size, slowing down the rate of replication. However, this size has not been directly measured to date nor has the effect of a large critical size been explored in detail in chemical kinetics models of aggregation.

There is limited data from cellular experiments that suggest possible values of this minimum size. Experiments using sonicated fibrils of different sizes, separated using a sucrose gradient, suggest that α‐synuclein aggregates of about 75–100 nm or less in length are not seed competent.^[9]^ This result is a combination of the probability of cell entry and probability of seeding and hence not a direct measurement of critical size. Super‐resolution imaging experiment in live cells, where cellular components may modify the thermodynamics of fibril formation, found a critical diameter of about 320 nm by fitting a first order phase transition for protein aggregate formation, presumed to be α‐synuclein aggregates.^[10]^ In contrast, data on prion protein aggregates, which may also contain co‐factors, show that the minimal diameter of an infectious particle is 17–27 nm.^[11]^

Super‐resolution imaging technology has already proved a useful tool in detecting and characterizing individual aggregate species with high specificity.^[12]^ In this work, we use super‐resolution fluorescence microscopy and atomic force microscopy to directly measure the size distributions of α‐synuclein aggregates formed in vitro and determine the critical size at which fibrillar aggregates are formed.

## Results and Discussion

**Figure 1:**
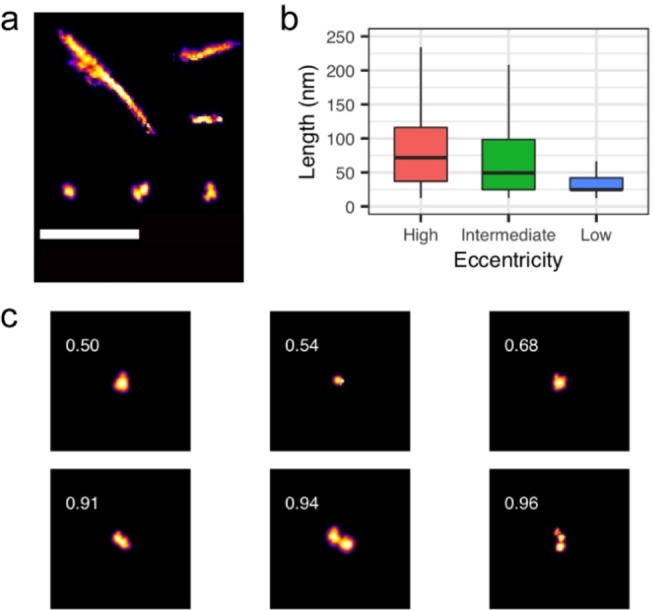
**Super‐resolution imaging of α‐synuclein aggregates with Aptamer DNA ‐PAINT**. a) Selected individual aggregates demonstrating varying size and morphology of α‐synuclein aggregates as they progress to mature fibrils (scale bar=1 μm). b) Length distributions of low, intermediate, and high eccentricity aggregates (defined as eccentricity <0.7, 0.7–0.95, >0.95, respectively) formed from homogenous monomer concentrations of 500 nM and 1 μM α‐synuclein between 7 and 22 days of incubation in Tris buffer at pH 7.5 with shaking at 37 °C (n=214,075). There was no difference in the length distribution between the 500 nM and 1 μM data so they were merged. c) Example AD‐PAINT images of aggregates with low and high eccentricities with the same scale as a).

Homogeneous 500 nM and 1 μM recombinant α‐synuclein monomer solutions were allowed to fully aggregate under shaking conditions at 37 °C. Aggregates were imaged with Aptamer‐DNA Point Accumulation for Imaging in Nanoscale Topography (AD‐PAINT) using a total internal reflection fluorescence (TIRF) microscope, as has been previously published.^[12]^ These super‐resolution images make it possible to distinguish morphological differences between the highly linear α‐synuclein fibrils and the comparatively more abundant, smaller, globular species which make up the vast majority of aggregates formed near physiological concentrations of α‐synuclein (1 μM) (Figure 1a). We used the eccentricity of the aggregates to distinguish fibrillar aggregates from globular aggregates (Figure 1 b and c). Over time, longer fibrillar aggregates with eccentricities greater than 0.95 form. The high eccentricity aggregates had a median length of 74 nm, while the aggregates with an eccentricity of less than 0.7 had a median length of 25 nm. Aggregates with eccentricities between 0.7 and 0.95 that could not be clearly classified had an intermediate median length of 54 nm. We noted that there was a clear transition between the low and high eccentricity aggregates at approximately 40 nm. Below this length the aggregates were more likely to have low eccentricity and above this length they were more likely to have high eccentricity.

**Figure 2:**
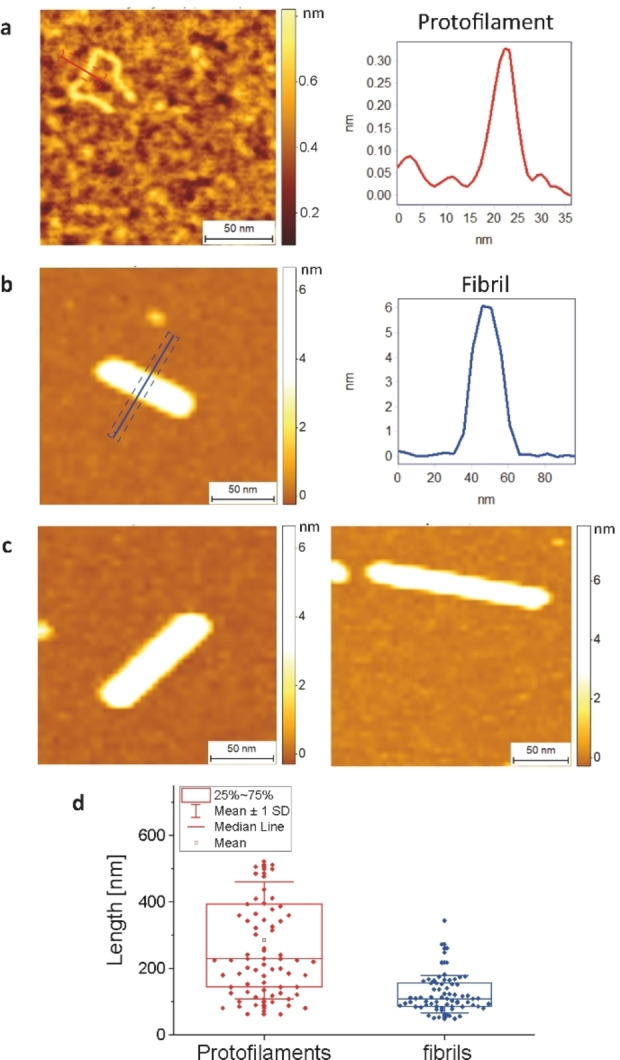
**High‐resolution 3D AFM imaging of α‐synuclein protofilaments and fibrils formed in Tris buffer**. Morphology maps showing a) a protofilament and its cross‐sectional diameter. b) Fibril and its cross‐sectional diameter. c) Fibrils of varying length. d) Single‐molecule statistical analysis and length distribution of synuclein protofilaments and fibrils.

To confirm this result we performed high‐resolution and phase controlled atomic force microscopy to acquire 3‐D morphology maps of α‐synuclein aggregation and single amyloid fibrillar species at the nanoscale. The aggregates were formed under the same conditions, but at 45 μM α‐synuclein concentration, to generate more fibrillar aggregates for imaging and single‐molecule statistical analysis without the need to dilute the sample, since dilution might alter the aggregate size distribution.^[11]^ In this case the sample is dried onto positive functionalized mica to image the negatively charged aggregates. The 3‐D maps at higher spatial resolution obtained from AFM allows us to distinguish intermediate protofilaments, with a cross‐sectional diameter of 0.3–4 nm, protofibrillar species which are long, flexible and thin, 2–5 nm diameter, from fibrils which are more mature and thicker, and have cross‐sectional diameter in the range of 6–10 nm.^[13]^ The length distribution of protofilaments, the first elongated species appearing in the aggregation pathway, showed an average length of 280±180 nm (Figure 2a–c). Because of their higher flexibility, protofilaments are more dynamic when imaged under solution using super resolution and hence will tend to appear spherical and less elongated. By contrast, mature fibrils appeared straight and showed a mean length of 120±60 nm and a minimal length of 50±10 nm, in excellent agreement with the super‐resolution imaging data. Importantly these data, based on measurement of 75 fibrils show a clear cut‐off at short lengths and are consistent with a transition from a long protofilament to a short fibril of 50±10 nm in length since we do not observe fibrils shorter than 40 nm (Figure 2d). This is entirely consistent with the results of the super‐resolution imaging experiments.

**Figure 3:**
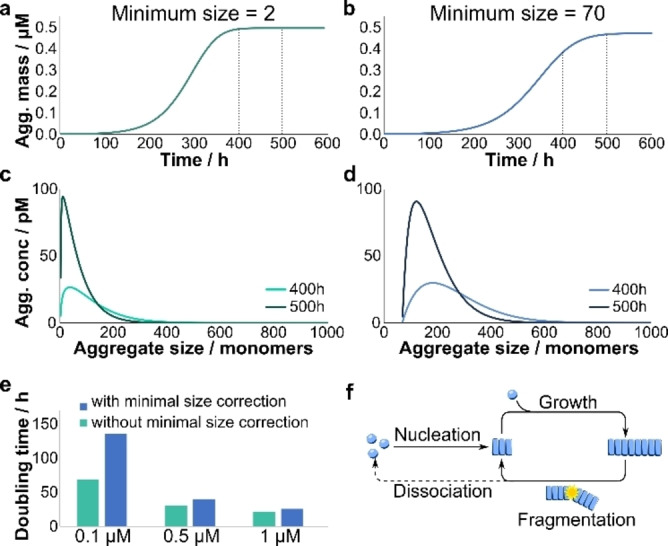
**Effect of minimum stable fibril size on aggregation kinetics and size distribution**. (a, b) The increase in aggregate mass over time, for a minimum stable fibril size of 2 (a) and 70 (b). (c, d) The size distribution at 400 h and 500 h for the respective cases. The corresponding times are marked by dashed lines in a and b. (e) Comparing the doubling time of aggregate replication when the minimum fibril size is taken into account and when it is neglected. While at 1 μM the doubling times are similar, at lower concentrations neglecting the loss of fibrils due to fragmentation below the minimum size results in doubling times that are significantly higher than the actual ones. (f) Schematic of the mechanism of aggregation. Initial fibrils are formed by primary nucleation, grow by elongation and multiply by fragmentation. Fragmentation into pieces smaller than the minimum stable size results in dissociation back into monomers.

To determine the importance of a minimum size for fibril formation we used a model which accounts for the observation that protofibrillar α‐synuclein must undergo a structural reconfiguration prior to growing into stable fibrillar aggregates. In the standard models of protein aggregation the minimum stable fibril size, also referred to as the nucleus size, is generally assumed to be negligibly small.[^6^, ^13^, ^14^] Therefore, any loss of fibril mass due to fragmentation into aggregates smaller than this minimum size is not an important effect in these descriptions. Here, we have explicitly considered the effect of a large minimum size and derived updated expressions for both the replication rate and the steady state average fibril size. For a negligible critical size, the replication rate is given by $\kappa =\sqrt{2k_{+}k_{f}m_{0}}$ , and the steady state average size, in numbers of monomers, by $\mu =\sqrt{2\frac{k_{+}m_{0}}{k_{f}}}$ , where *k*_*+*_ is the elongation rate constant, *k_f_* is the fragmentation rate constant and *m_0_* is the monomer concentration at the beginning of the reaction. When we take into account that fragmentation can generate fibrils smaller than the critical size, which will then dissociate, we find that the replication rate is given approximately by

(1) $$\bar{\kappa }=\kappa -k_{f}n_{c}\;\mathrm{and}\;\bar{\mu }=\mu +n_{c},$$

where *n_c_* is the minimum stable fibril size (the bar denoting quantities when the minimum size is taken into account). A detailed derivation can be found in the methods. This approximation is accurate for critical sizes up to the steady state length μ , but breaks down for larger values. It can easily be shown that the effect of critical size becomes important for both the replication rate and the steady state size when *n_c_* approaches in magnitude the steady state length *μ*. In other words, the effect of a minimum stable fibril size can be ignored if it is much shorter than the average size of the aggregates. The fact that the minimum size observed in our experiment is close to the average size indicates that indeed the minimum size, in addition to the rate constants of fragmentation and growth, is a key factor in determining the speed at which α‐synuclein fibrils can replicate. We illustrate the importance of accounting for this factor in Figure 3 where taking account of the critical size reduces the doubling time by a factor of two for 0.1 μM α‐synuclein. The magnitude of this effect increases the lower the α‐synuclein concentration.

It is not possible to obtain the critical aggregate size from a chemical kinetic analysis of an aggregation reaction measured using a dye like Thioflavin T, since high‐resolution length distributions are required. Therefore, the critical size has not been determined for any aggregation reaction to date. Our results show that the critical length for formation of α‐synuclein mature fibrils is about 40 nm. Aggregates with a smaller length and containing a smaller number of monomers appear to not be stable in this fibrillar form and instead exist as longer, thinner protofilaments and protofibrils and as smaller spherical aggregates, which contain fewer monomers.

We can obtain a lower bound on the number of monomers in the minimum size fibril of 40 nm length and diameter of 6 nm using the standard density of a folded protein of 1.35 kg ⋅ L^−1^. This gives an estimate of the critical size as 64 monomers. We can also estimate the number of monomers in a protofilament, since we previously demonstrated that the protofilaments in Figure 2a result from the direct assembly of α‐synuclein monomers into a linear chain.^[13]^ If we assume that there is one monomer per 4 nm of a single‐strand protofilament (diameter partially folded monomer),^[14]^ we obtain an average number of 70 monomers per protofilament and a range of protofilament sizes between 10 and 130 monomers. Thus, a significant subpopulation of protofilaments contains enough monomers to form a stable fibril. Therefore, in agreement with our previous kinetic analysis,^[6]^ the conversion from protofilament to fibril could be a unimolecular process. Previous work established the rate of conversion to be on the order of 0.1 h^−1^, under the assumption that all measured oligomeric species are taking place in that conversion reaction.^[6]^ However, our findings suggest that, in fact, only a subset of the oligomeric population is large enough to convert to stable fibrils, thus the concentration of conversion‐competent species was overestimated and consequently the conversion rate underestimated. Based on our previous measurements of the oligomer size distribution,^[6]^ we conclude that the conversion rate is at least 2 orders of magnitude higher than previously estimated, giving rapid conversion times of a few minutes or less.

To be an effective seed, a fibril would need to be able to produce two new fibrils that are larger than the critical length, so the seed effectiveness will depend on the balance of growth and fragmentation rates as well as the size of the seed. Altered rates in cells may explain why the observed minimal effective seed sizes under those conditions are somewhat larger than the minimal stable fibril size we observe here.^[9]^ Previous work has estimated the critical radius for aggregate formation by what is believed to be α‐synuclein to be about 160 nm, giving a diameter of 320 nm, in cells, suggesting that a critical transition occurs in living cells.^[10]^ The value of 320 nm obtained in cells is significantly larger than the value obtained in our study in solution and may represent a different transition that occurs for larger aggregates in the cellular environment. Fibrillar aggregates may also be significantly destabilised in the cellular environment, possibly due to post‐translational modifications. Additionally, the labels used in the cellular experiments may destabilise the fibrillar aggregates. Finally, smaller aggregates may also be more susceptible to cellular removal processes, skewing the size distribution. Thus, experiments imaging the aggregates in cells without labels would be needed to help determine the origin of this difference.

The role of prion‐like spreading in causing aggregation in neighbouring neurons in neurodegenerative disease is a subject of intense research and, in particular, it is not clear how potential fibril seeds are formed. However, it is clear that cells can spontaneously form aggregates under conditions of increased protein expression or cell stress. Our result combined with our recent study of seeding in cells^[15]^ suggests that once aggregates larger than the critical size are formed in the cytosol they can convert to the fibrillar form and replicate by elongation and fragmentation very rapidly. Hence, the cell must continuously remove or secrete aggregates to prevent them growing by monomer addition to 70‐mers, since aggregates of this size can convert within minutes into fibrils. In our recent study of SH5Y cells we observed no accumulation of aggregates in the cell but significant secretion of small aggregates of about 35 nm in size under basal conditions which increased when the cell were seeded.^[15]^ Hence, preventing aggregates from reaching their critical size appears to be a key cellular protective mechanism. We speculate that formation of aggregates larger than 70‐mers, 40 nm in size, may be the tipping point that overwhelms the protein homeostasis of the cellular machinery. Conversely, preventing formation of fibrillar aggregates of this size or finding ways to increase the critical size may be a general strategy to reduce or prevent aggregate induced cell death.

## Conclusion

In summary, we have shown that an aggregate of α‐synuclein will only form fibrils when it contains more than 70 monomers and this value determines the minimum size of an α‐synuclein “prion”. Aggregates with fewer than 70 monomers have a different structure and properties from mature fibrils. Our work defines a simple framework to determine this critical size for other proteins associated with neurodegenerative disease, such as beta amyloid and tau, and to determine the effect of mutations or different conditions on this length. Our modelling shows that this critical size is an important parameter that governs the rate of aggregate replication, together with the elongation and fragmentation rate constant, and this large critical size for α‐synuclein may contribute to its slow spreading observed in vivo.

## Experimental Section

Please see the Supporting Information for detailed experimental procedures.

## Author Contributions

SES and DRW performed the super‐resolution imaging. SES performed the analysis of super‐resolution images. FSR performed the AFM experiments and analysis. GM performed the modelling. EH performed the eccentricity analysis. All authors contributed to the writing of the paper.

## Conflict of interest

The authors declare no conflict of interest.

## Supporting information

As a service to our authors and readers, this journal provides supporting information supplied by the authors. Such materials are peer reviewed and may be re‐organized for online delivery, but are not copy‐edited or typeset. Technical support issues arising from supporting information (other than missing files) should be addressed to the authors.

Supporting Information
