# Supplementary material for: Alpha Synuclein only Forms Fibrils In Vitro when Larger than its Critical Size of 70 Monomers
Source: Chembiochem. 2021 Aug 24;22(19):2867–71. doi: 10.1002/cbic.202100285 (PMC8518629; doi:10.1002/cbic.202100285)
Supplement: Supplementary file 1 — Supporting Information [file CBIC-22-2867-s001.pdf]

ChemBioChem

Supporting Information

Alpha Synuclein only Forms Fibrils In Vitro when Larger than its Critical Size of 70 Monomers

Santiago Enrique Sanchez, Daniel R. Whiten, Georg Meisl, Francesco Simone Ruggeri, Eric Hidari, and David Klenerman*

Methods

Super-resolution imaging

Recombinant wild-type monomeric α-synuclein was a gift from the late Professor Sir Christopher Dobson. The synuclein was centrifuged at 270,000 g and 4°C in order to remove any fibrillar aggregates. The concentration of protein in the supernatant was determined using a bicinchoninic acid (BCA) assay (Thermo-Fisher Scientific) and then diluted in 1X Tris buffer (5.0 mM Tris, 1 mM EDTA, 10 mM $MgCl_2$) , pH 7.5; 0.02 µm filtered, Anotop25, Whatman) to one of two starting concentrations (500 nM, and 1 µM) of monomeric α-synuclein . Each aliquot was then left to aggregate at 200 RPM and 37°C following the addition of 0.1% NaN_3) to prevent bacterial contamination. All aggregations were prepared in triplicate. One aliquot of 70 µM α-synuclein was prepared in the same way and monitored under shaking conditions for fibril formation over 48 hours in order to verify the aggregation competency of the monomeric α-synuclein in the stock solution, effectively acting as a positive quality control selected for its reliable kinetics[1] .

TIRF microscopy was utilized to perform AD PAINT as previously reported to generate super-resolution images of aggregates[2].

AD PAINT exploits the transient binding of an imaging strand — a single-stranded, 9 bp sequence of fluorophore-conjugated CCAGATGTAT-CY3B DNA — to a single-stranded docking strand attached to an aptamer evolved to specifically bind aggregates of α-synuclein (GCCTGTGGTGTTGGGGCGGGTGCGTTATCTACATA)[12,16]. Glass microscope slides (0.13 mm thickness, round, 50 mm diameter) were first dusted via nitrogen stream before being cleaned with Argon plasma (PDC-002, Harrick Plasma) for 1 h. A multi-well chambered coverslip (CultureWell CWCS-50R-1.0, 50 channels) was then attached to the surface in two layers after being cut in half. Tween-20 solution (1% in 0.02 µm filtered PBS, Anotop25, Whatman) was pipetted into each well and allowed to coat the surface for 1 h in order to passivate the glass-bottomed surface of the well and minimize non-specific binding of the aptamer and imaging strand to the surface. Following passivation, the Tween-20 solution was washed off and a solution containing the α-synuclein sample of interest was introduced into each well and allowed to coat the surface for 10 min. The sample solution was then replaced with an imaging solution containing 100 nM aptamer-docking strand, 1 nM of imaging strand, and 5 µM of the beta-sheet binding dye Thioflavin T (Sigma-Aldrich; 0.02 µm filtered, Anotop25, Whatman) is introduced into each well. Thioflavin T is a ß-sheet binding dye commonly used to detect amyloid structures and was used here to confirm the presence of aggregates and fibrils prior to single-molecule image acquisition. The wells are then sealed with a second plasma-cleaned microscope slide. Given that the evanescent field generated through TIRF only penetrates approximately 100 nm into the sample, only the fluorescence from imaging strands bound to the aptamer-docking strand on the surface of the slide is captured by the camera. For each field of view, 4000 frames at 50 ms exposure were captured while exciting the CY3B imaging strand with the 561 nm laser followed by 100 frames at 50 ms exposure while exciting the Thioflavin T dye with the 405 nm laser. Six fields of view were captured in succession for each imaged well using a custom script (MicroManager).

Individual fluorescence events captured in each frame were localized and super-resolved using the PeakFit function of the GDSC (University of Sussex) Single Molecule Light Microscopy (SMLM) package in ImageJ utilizing a signal threshold of 30 and a precision of 10 nm. Oligomers and fibrils were identified utilizing the Density Based Spatial Clustering of Applications with Noise (DBSCAN) algorithm in Python (sklearn v0.18.1, epsilon = 3 pixels, minimum point threshold of 10), one of the most commonly used clustering algorithms currently employed for scientific applications[3]. Furthermore, dimensional analysis of each super-resolved cluster was carried out using a Python skeletonizing protocol allowing us to determine the length of each aggregate as previously described[4]. In brief, clusters of fluorescence events containing at least 10 events were grouped by proximity until no more neighboring clusters were detected beyond a radius of 3 pixels. The length and eccentricity measurements of each cluster are automatically generated following clustering. Code is available upon request.

Atomic force microscopy (AFM) experiments

A 45 μM monomeric (>95%) α-synuclein filtrated solutions in a 50 mM TRIS-buffer, NaCl 150 mM, and pH 5.5 and 7.5, were incubated at 37 °C in an sealed Eppendorf tube to avoid contamination and evaporation. The sample was shaken for 10 days. The experiments were repeated in triplicates.

Atomic Force Microscopy was performed on positively functionalized mica substrates. We cleaved the mica surface and we incubated it for 1 minute with 10 μl of 0.5% (v/v) 3-aminopropyl-triethoxysilane (APTES, from SIGMA) in Milli-Q water. Then, the substrate was rinsed three times with 1 ml of Milli-Q water and dried by gentle stream of nitrogen gas. Finally, for each sample, an aliquot of 10 μl of the solution was deposited on the positively functionalized surface. The droplet was incubated for 10 minutes, then rinsed by 1 ml of Milli-Q water and dried by the gentle stream of nitrogen gas. The preparation was carried out at room temperature. AFM maps were realized by means of a NX10 (Park Systems) operating in non-contact mode and equipped with a silicon tip (PPP-NCHR, 5 Nm^{-1}) with a nominal radius <10 nm. Image flattening, analysis of cross-sectional dimensions and single aggregate tracing was performed by SPIP (Image metrology) software, while data were plotted by OriginPRO.

Calculation of fibril length distributions

The time evolution of the aggregate length distribution was obtained by numerical integration of the master equation, using a fourth order Runge-Kutta algorithm. The master equation is a set of infinitely many differential equations, given by:

$$\frac{df(t,j)}{dt} = 2k_+m(t)\big(f(t,j-1) - f(t,j)\big) + 2\,k_{off}\big(f(t,j+1) - f(t,j)\big) - k_-(j-1)f(t,j) + 2\,k_- \sum_{i=j+1}^{\infty} f(t,i)$$

for $j>n_{min}$ and

$$\frac{df(t,j)}{dt} = -2k_+m(t)\big(f(t,j)\big) + 2\,k_{off}\big(f(t,j+1) - f(t,j)\big) - k_-(j-1)f(t,j) + 2\,k_- \sum_{i=j+1}^{\infty} f(t,i) + k_n m(t)^{n_c}$$

for $j>n_{min}$.where f(t,j) is the concentration of species of size j at time t, m(t) is the monomer concentration at time t, k_+, k_{off}, k_- and k_n are the rate constants of elongation, depolymerization, fragmentation and primary nucleation respectively, n_c is the reaction order of primary nucleation and n_{min} is the minimum stable fibril size, which is also the size of fibril produced by primary nucleation. Note that in previous descriptions[5] the minimum stable fibril size and the reaction order of primary nucleation were the same parameter, n_c. To allow for more flexibility we here introduce two separate parameters for the reaction order of primary nucleation and the minimum stable fibril size. In order to be able to integrate this infinite set of equations numerically, we chose a maximum fibril size j_{max} = 2000, governed by the equation:

$$\frac{df(t,j)}{dt} = 2k_+m_0\big(f(t,j-1)\big) - 2\,k_{off}\big(f(t,j)\big) - k_-(j-1)f(t,j)$$

To minimize any effects due to this finite maximum size, we monitored the $f(t,j_{max})$, ensuring that only a negligibly small concentration of fibrils of the maximum length was present.

Approximate analytical expressions for average size and replication rate

As detailed in Cohen et al[5], the moment equations describing the time evolution of fibril number P(t) and fibril mass M(t) are given by:

$$\frac{dP(t)}{dt} = k_-(M(t) - (2n_c - 1)P(t)) + k_n m(t)^{nc}$$

$$\frac{dM(t)}{dt} = \left(2k_+ m(t) - k_- n_c(n_c - 1)\right)P(t)$$

where the parameters are as defined above. Linearizing these equations approximating the monomer concentration as constant, m(t) ~ m_0, which is a valid approximation for early times, produces a system of first order ordinary differential equations, which, for unseeded initial condition, can be solved to give:

$$M(t) = \frac{k_n m_0^{nc}}{k_- n}\left(e^{\frac{k_- t(1-2n_c)}{2}}\left(Cosh(\kappa t) + \frac{k_-(2n_c - 1)}{2\kappa}Sinh(\kappa t)\right) - 1\right)$$

where κ is the replication rate when the critical size is negligible. The approximate replication rate when the critical size is taken into account, $\bar{\kappa}$, can be identified by considering the exponential growth part as $\bar{\kappa} = \kappa - k_f n_c$. Similarly, an approximate steady state average length, which is given by $\mu = \frac{M(t)}{P(t)}$, can be obtained as $\bar{\mu} = \mu + n_c$. Clearly the approximation for $\bar{\kappa}$ breaks down when the critical size n_c becomes too large, as the replication rate must be a positive number. For less extreme values of n_c these general but approximate expressions agree well with the values obtained from numerical integration with specific parameters.

References

[1] M. Iljina, G. A. Garcia, M. H. Horrocks, L. Tosatto, M. L. Choi, K. A. Ganzinger, A. Y. Abramov, S. Gandhi, N. W. Wood, N. Cremades, C. M. Dobson, T. P. J. Knowles, D. Klenerman, *Proceedings of the National Academy of Sciences* **2016**, *113*, E1206-E1215.

[2] D. R. Whiten, Y. K. Zuo, L. Calo, M. L. Choi, S. M. De, P. Flagmeier, D. C. Wirthensohn, F. Kundel, R. T. Ranasinghe, S. E. Sanchez, D. Athauda, S. F. Lee, C. M. Dobson, S. Gandhi, M. G. Spillantini, D. Klenerman, M. H. Horrocks, *Chembiochem* **2018**, *19*, 2033-2038.

[3] K. Tsukakoshi, K. Abe, K. Sode, K. Ikebukuro, *Anal Chem* **2012**, *84*, 5542-5547.

[4] M. Ester, H. P. Kriegel, J. Sander, X. Xiaowei, *A density-based algorithm for discovering clusters in large spatial databases with noise*, AAAI Press, Menlo Park, CA (United States), **1996**.

[5] S. I. Cohen, M. Vendruscolo, M. E. Welland, C. M. Dobson, E. M. Terentjev, T. P. Knowles, *J Chem Phys* **2011**, *135*, 065105.
